# CD21^lo/−^CD27^−^IgM^−^ Double-Negative B Cells Accumulate in the Joints of Patients With Antinuclear Antibody-Positive Juvenile Idiopathic Arthritis

**DOI:** 10.3389/fped.2021.635815

**Published:** 2021-04-16

**Authors:** Johannes Dirks, Jonas Fischer, Gabriele Haase, Annette Holl-Wieden, Christine Hofmann, Hermann Girschick, Henner Morbach

**Affiliations:** ^1^Pediatric Immunology, University Children's Hospital, Würzburg, Germany; ^2^Pediatric Rheumatology and Osteology, University Children's Hospital, Würzburg, Germany; ^3^Vivantes Children's Hospital, Berlin, Germany

**Keywords:** juvenile idiopathic arthritis, B cells, antinuclear antibodies, synovial fluid, double negative B cells

## Abstract

Juvenile idiopathic arthritis (JIA) encompasses a heterogeneous group of diseases. The appearance of antinuclear antibodies (ANAs) in almost half of the patients suggests B cell dysregulation as a distinct pathomechanism in these patients. Additionally, ANAs were considered potential biomarkers encompassing a clinically homogenous subgroup of JIA patients. However, in ANA+ JIA patients, the site of dysregulated B cell activation as well as the B cell subsets involved in this process is still unknown. Hence, in this cross-sectional study, we aimed in an explorative approach at characterizing potential divergences in B cell differentiation in ANA+ JIA patients by assessing the distribution of peripheral blood (PB) and synovial fluid (SF) B cell subpopulations using flow cytometry. The frequency of transitional as well as switched-memory B cells was higher in PB of JIA patients than in healthy controls. There were no differences in the distribution of B cell subsets between ANA- and ANA+ patients in PB. However, the composition of SF B cells was different between ANA- and ANA+ patients with increased frequencies of CD21^lo/−^CD27^−^IgM^−^ “double negative” (DN) B cells in the latter. DN B cells might be a characteristic subset expanding in the joints of ANA+ JIA patients and are potentially involved in the antinuclear immune response in these patients. The results of our explorative study might foster further research dissecting the pathogenesis of ANA+ JIA patients.

## Introduction

Juvenile idiopathic arthritis (JIA) is the most common rheumatic disease of childhood and affects joints and frequently eyes (1). Although the pathogenesis is still unexplained in detail, the occurrence of autoantibodies (e.g., antinuclear antibodies, ANA) in a significant proportion of patients suggests involvement of autoreactive B cells (2, 3).

B cell differentiation is tightly controlled and several tolerance mechanisms are in place that restrain the development and/or activation of autoreactive B cells (4, 5). After encounter of antigens, mature naïve B cells differentiate into memory B cells and plasma cells. Memory B cells in humans express CD27 as a characteristic marker and can further be separated according to their expressed immunoglobulin isotype into IgD^+^IgM^+^ non-switched memory (NSM) and IgD^−^IgM^−^ switched memory (SM) B cells (6). Besides plasma cells and memory B cells, CD21^lo/−^ B cells seem to constitute an effector B cell population that is expanded in several autoimmune but also infectious diseases (7–9). CD21^lo/−^ B cells are characterized by expression of the transcription factor T-bet as well as the chemokine receptors/integrin CXCR3 and CD11c. They accumulate in inflamed tissues and seem to be enriched in autoreactive B cell clones (7, 8, 10). Since this phenotype may appear in developmentally different B cell subsets, CD21^lo/−^ B cells rather constitute a particular B cell effector state than a distinct B cell subset (9). Nevertheless, CD21^lo/−^ B cells that have undergone isotype switch (IgD and IgM negative) but did not upregulate the B cell memory marker CD27 have been described as a distinct B cell subset called “atypical memory” or “double negative” (DN) B cells. Those cells may be expanded in autoimmune diseases (9, 11, 12).

Several observations suggest that the otherwise tightly controlled process of B cell differentiation and tolerance induction may be disturbed in JIA patients. The immunoglobulin repertoire of peripheral blood B cells of JIA patients shows features of defective B cell tolerance (13). Additionally, switched memory B cells are expanded in the peripheral blood (PB) of oligo- and poly-JIA patients with higher expansion rates in patients with early-onset disease (14). Oligoclonal accumulation of memory B cells in the synovial fluid (SF) of JIA patients suggests antigen-driven activation of B cells within the inflamed tissues potentially triggered by local antigens (15). Indeed, indirect signs of aberrant B cell activation such as plasma cell infiltration and/or lymphoid neogenesis in synovia or iris tissue seem to correlate with ANA positivity in JIA patients (16–18). Hence, the tightly controlled processes of B cell differentiation and tolerance induction seem to be disturbed in JIA patients at several sites. However, whether these alterations are associated with the development of autoantibodies or rather reflect unspecific changes due to chronic inflammation is unknown. Also, the composition of the B cell compartment in the inflamed tissues of ANA+ JIA patients is not elucidated in detail yet. We therefore aimed at analyzing the distribution and phenotype of PB and SF B cell subsets in JIA patients and at dissecting differences between ANA positive and negative patients.

## Materials and Methods

### Patients

All JIA patients included in this cross-sectional study fulfilled the ILAR classification criteria for JIA. The inclusion criteria were active disease at time point of analysis (defined by swelling or limitation of motion with pain or tenderness), and classification within subgroup of oligoarthritis, rheumatoid-factor-negative polyarthritis, or psoriasis arthritis and ANA titer determined by immunofluorescence tests. We included consecutive patients with JIA who presented to our outpatient clinic and had blood drawn due to clinical indication. JIA patients were recruited into two independent studies: the first study aimed at analyzing the distribution of peripheral blood (PB) B cell populations (cohort 1), whereas the second study aimed at analyzing the distribution of synovial fluid (SF) B cell populations (cohort 2). Recruitment of patients into the two studies took place independently and at different time points (cohort 1: 2009–2010, cohort 2: 2011–2018). JIA patients were classified according to their JIA subgroup as well as to the presence of ANAs. In accordance with other studies (19–21), an ANA titer of ≥1:160 was regarded as positive and <1:80 as negative; JIA patients with an ANA titer of 1:80 were classified as “intermediate” and for reasons of comparability excluded from analysis when comparing ANA+ and ANA- JIA patients (19–21). All patients were followed at University Children's Hospital Würzburg. Signed informed consent was obtained by the legal representatives. The study was reviewed by the Research Ethics Committee of the University of Würzburg (299/17) and conducted in accordance with the Declaration of Helsinki.

### Sample Preparation

SF and/or PB samples were collected in EDTA tubes. Mononuclear cells were isolated by Ficoll density-gradient centrifugation, as previously described (15). Mononuclear cells were resuspended in FCS 10% DMSO and stored in liquid nitrogen until use.

### Flow Cytometry

After thawing and washing mononuclear cells were stained in 1 × PBS 0.5% BSA with appropriate antibodies at 4°C for 30 min. Flow cytometry data of cohort 1 was acquired on a FACSCalibur. Since a FACSCanto II was available at the time point of the analysis of cohort 2, we preferred to perform the analysis on this instrument. All flow cytometry data was analyzed with FlowJo version 10 (Tree Star). The following antibodies were used—cohort 1: CD19 APC (HIB19), CD24 FITC (ML5), CD38 PE (HIT2), CD45 PerCP (2D1), IgD PE (IA6-2), CD27 FITC (M-T271) (all from BD Biosciences) and CD21 FITC (clone 1F8, Dako); cohort 2: CD19 APC-Cy7 (HIB19), CD20 PE-Cy7 (2H7), CD21 APC (Bu32), CD22 FITC (S-HLL-1), CD27 PerCP-Cy5.5. (M-T271), CD38 PE (HIT2), IgM Brilliant Violet 421 (MHM88), IgD PE (IA6-2), T-bet Brilliant Violet 421 (IT2.2), FAS Brilliant Violet 421 (Dx2), IgG PE (HP6017) (all from BioLegend), IgA FITC (IS11-8E10) (Miltenyi Biotec), and CD11c PE (3.9) (ebioscienes).

### Statistical Analysis

Statistical analysis was performed using Prism 8.0 (GraphPad). Hierarchical cluster analysis and heatmap visualization were performed using ClustVis. Data is expressed as scattered individual values and mean ± SD. Paired and unpaired Student's *t*-tests were used for comparison of data sets with two or more continuous variables, respectively. The Chi-square test was used to test relationships between categorical variables. A *p*-value < 0.05 was considered as statistically significant.

## Results

### Study Population

The patients' demographic and clinical data are summarized in Supplementary Tables 1, 2. All patients were Caucasian. The PB B cell compartment was studied in 45 JIA patients (cohort 1, Supplementary Table 1). To avoid batch effects, the flow cytometric analysis of PB B cells in JIA patients was performed in the time period 2009–2010 in parallel to a cohort of healthy control individuals from which reference values for PB B cell subsets had been established (22). For each JIA patient from cohort 1, we matched one of these healthy control individuals for sex and age (mean age difference 0.21 years, maximum age difference 0.6 years). These individuals served as healthy control group. After having analyzed the distribution of PB B cell subsets in cohort 1, a second cohort of 43 JIA patients undergoing joint puncture for intra-articular steroid injection was included in this study for the analysis of the SF B cell compartment (cohort 2, Supplementary Table 2; recruitment 2011–2018, flow cytometric analysis 2017–2019). From five of these patients, additional matched PB samples from the same date were available for direct comparison. Nine patients were included in both cohorts; however, biomaterials from these patients (PB, cohort 1 or SF, cohort 2) have been obtained at different time points and were therefore excluded from direct comparison. Most included patients could be assigned to the subgroup of oligoarthritis and about half of the patients were ANA positive (Supplementary Tables 1, 2). As reported before, the group of ANA+ JIA patients tended to have an earlier disease onset with a female preponderance (19, 20).

### Increased Frequencies of Transitional and Switched Memory B Cells in the Peripheral Blood of JIA Patients

**Figure 1:**
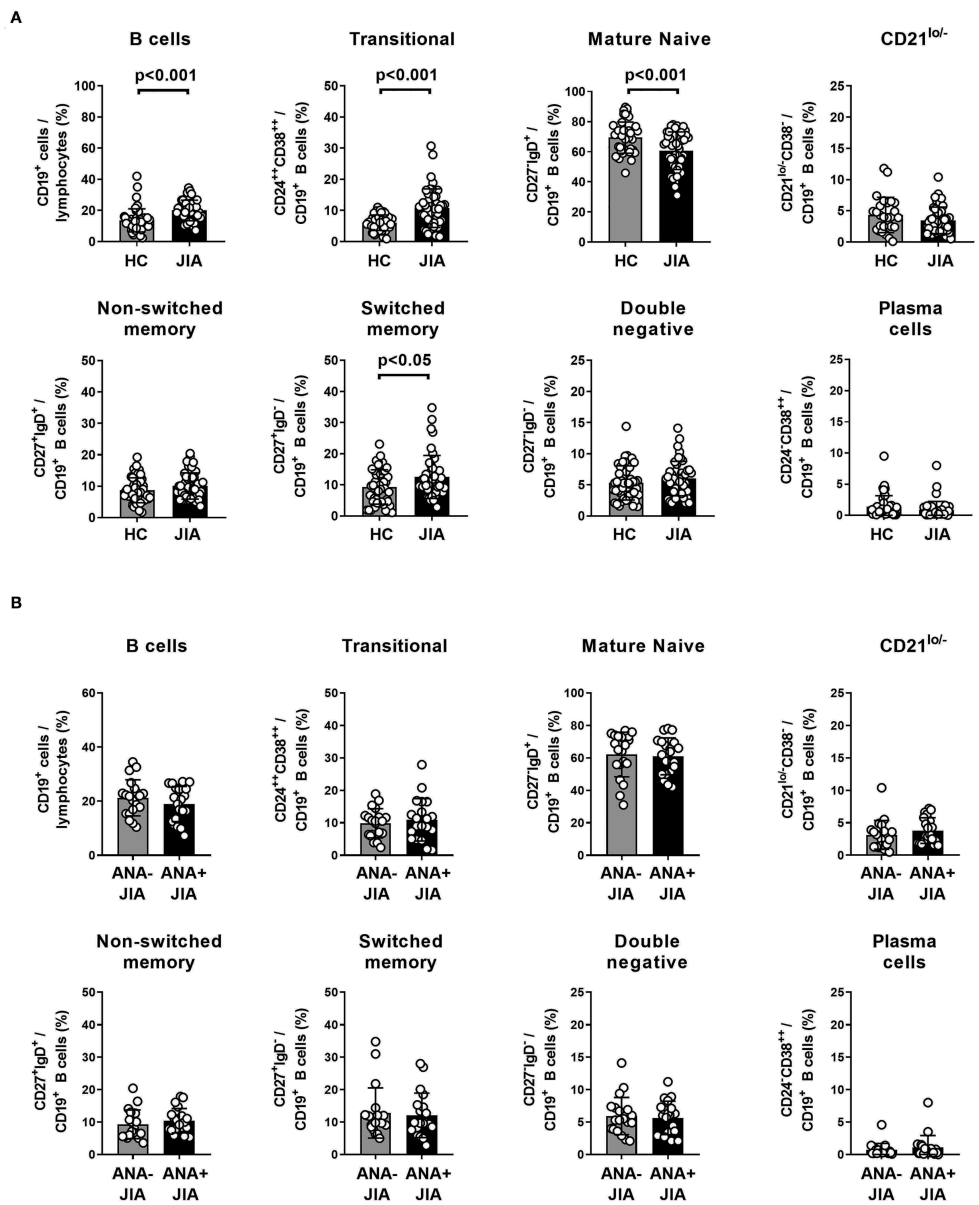
Distribution of peripheral blood B cell population in ANA-positive and ANA-negative JIA patients. **(A)** Frequency of peripheral blood CD19^+^ B cells within lymphocytes as well as different B cell populations within CD19^+^ B cells in a cohort of JIA patients (*n* = 45) and same number of age- and sex-matched healthy control (HC) individuals. Bars represent mean frequency with standard deviation and circles individual data points. **(B)** Frequency of these B cell populations from JIA patients was compared between ANA+ and ANA– patients. Groups were compared using unpaired Student's *t*-test.

To search for potential JIA associated deviations of B cell differentiation from normal we first analyzed the distribution of PB B cell subsets within a cohort of 45 JIA patients. We compared that data to age- and sex-matched healthy control individuals derived from an established reference cohort generated in our laboratory using a gating strategy as outlined previously (Supplementary Figure 1) (22). The frequency of total CD19^+^ B cells within PB lymphocytes was higher in JIA patients than in healthy individuals (Figure 1A). Additionally, JIA patients displayed alterations in the composition of the PB B cell compartment: the frequency of CD24^++^CD38^++^ transitional B cells was higher in JIA patients whereas that of mature naïve B cells was lower in JIA patients (Figure 1A). Also, JIA patients displayed higher frequencies of switched-memory B cells within the PB B cell compartment (Figure 1A). To assess whether the presence of ANAs in particular is associated with the observed changes in the PB B cell compartment, we compared the data of ANA+ and ANA– JIA patients within this cohort. However, we could not observe differences in the frequency of any of the analyzed PB B cell subsets between ANA+ and ANA– JIA patients (Figure 1B). We further plotted the data against age at sampling to correct for any age bias between both subgroups that might exist since ANA+ JIA patients are younger at disease onset and maturation of the PB B cell compartment follows an age-dependent sequence (19, 20, 22). For none of the analyzed B cell subsets, we detected significant differences in the proportion of data points outside the reference ranges between ANA+ and ANA– patients (Supplementary Figure 2). Thus, the deviations from normal observed in the composition of the PB B cell compartment were present in ANA+ as well as ANA– JIA patients (Supplementary Figure 2). Hence, expansion of transitional and switched-memory B cells characterized the PB B cell pool in JIA patients and these changes were not associated with the presence of ANAs.

### CD21^lo/–^ Switched Memory and Double-Negative B Cells as Well as Plasma Cells Dominate the Synovial Fluid B Cell Compartment

**Figure 2:**
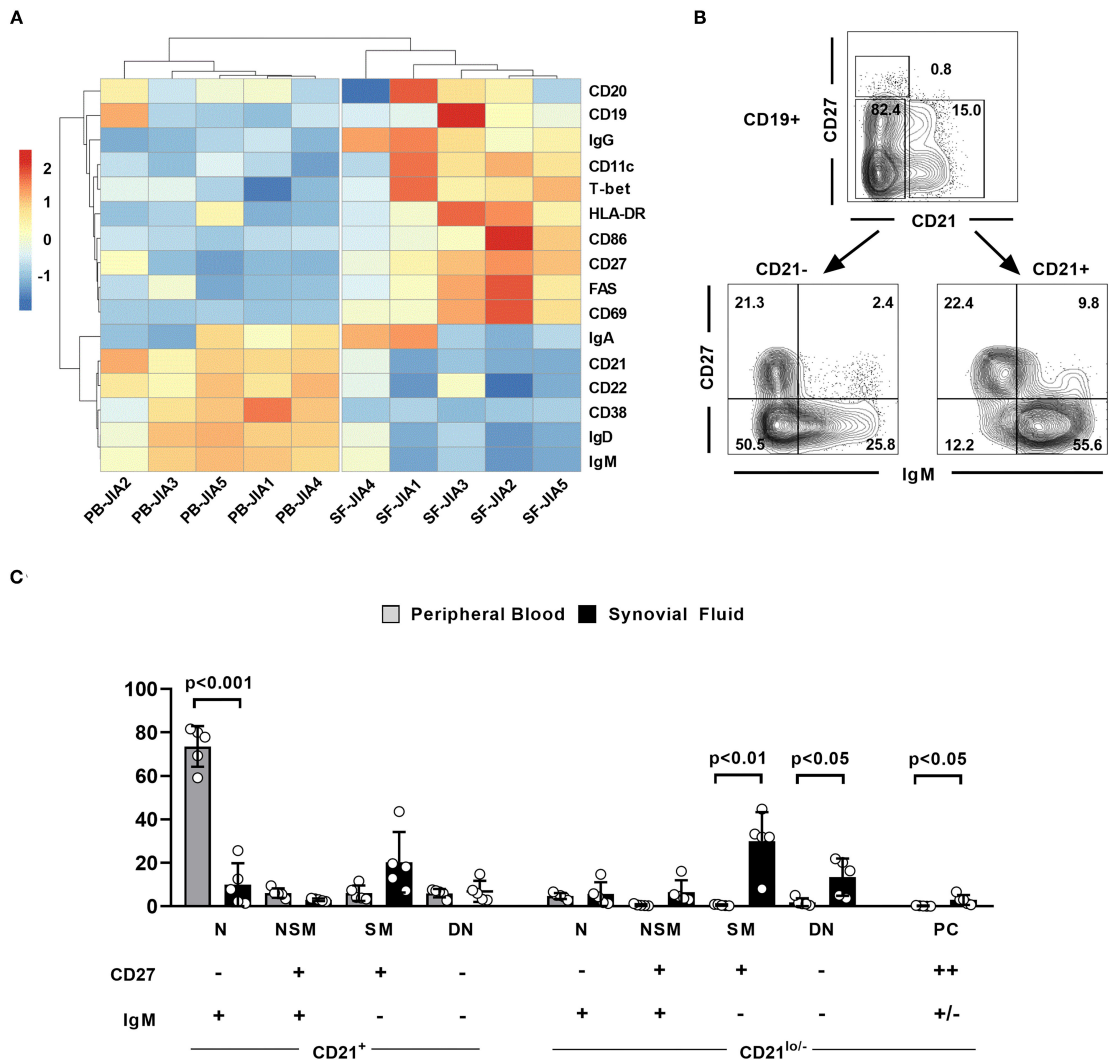
The extended phenotype of synovial fluid B cells in JIA patients. **(A)** Hierarchical cluster analysis of mean fluorescence intensities of different markers in CD19^+^ peripheral blood (PB) and synovial fluid (SF) B cells from five JIA patients assessed by flow cytometry. **(B)** Flow cytometric gating strategy of different SF B cell populations. **(C)** Distribution of different B cell populations within CD19^+^ PB and SF B cells of five JIA patients. Groups were compared using paired Student's *t*-test. Bars represent mean frequency with standard deviation and circles individual data points. N, Naïve; NSM, non-switched memory; SM, switched memory, DN, double negative; PC, plasma cells.

The alterations observed in the PB B cell compartment of JIA patients might not reflect the local situation at the site of inflammation, especially since naïve B cells are scarce in the joints of JIA patients and activated memory B cells prevail the SF B cell compartment (15, 23). We therefore aimed at characterizing the SF B cell compartment in more detail and comparing its composition between ANA+ and ANA– JIA patients. Sufficient biomaterials from five matched PB and SF samples were available for a detailed comparison of the extended B cell phenotype between both compartments. SF B cells were clearly separated from PB B cells by hierarchical cluster analysis of this data set (Figure 2A). In detail, SF CD19^+^ B cells displayed the phenotype of IgG+ class-switched and activated memory B cells whereas the extended B cell phenotype of PB B cells resembled non-switched and resting B cells (Figure 2A). Moreover, upregulation of CD11c and T-bet as well as downregulation of CD21 in the SF B cell compartment resembled an expression signature characteristic for CD21^lo/−^ B cells (Figure 2A) (9). Due to the distinct phenotype of SF B cells, we slightly adapted our gating strategy used for the analysis of PB B cells now focusing on effector B cell populations present in the SF. In detail, we aimed at quantitatively comparing the distribution of defined SF and PB B cell subsets according to their expression of CD21, IgM, and CD27 (Figure 2B). We used IgM instead of IgD to accurately delineate switched-memory (SM) B cells from IgM-only memory B cells; the latter do express IgM but not IgD and might falsely be identified as SM B cells when using IgD. Whereas, CD21^+^CD27^−^IgM^+^-naïve (N) B cells dominated within the PB B cell compartment, the frequency of CD21^lo/−^CD27^+^IgM^−^ SM and CD21^lo/−^CD27^−^IgM^−^ DN B cells B cells was significantly higher in the SF than in the PB B cell pool (Figure 2C). Additionally, the frequency of CD27^++^ plasma cells was slightly higher in SF than in PB (Figure 2C). Thus, activated SM and DN B cells that show the phenotype of activated CD21^lo/−^ B cells preferentially accumulate in the joints of JIA patients.

### Expansion of CD21^lo/–^ Double-Negative B Cells in the Joints of ANA+ JIA Patients

**Figure 3:**
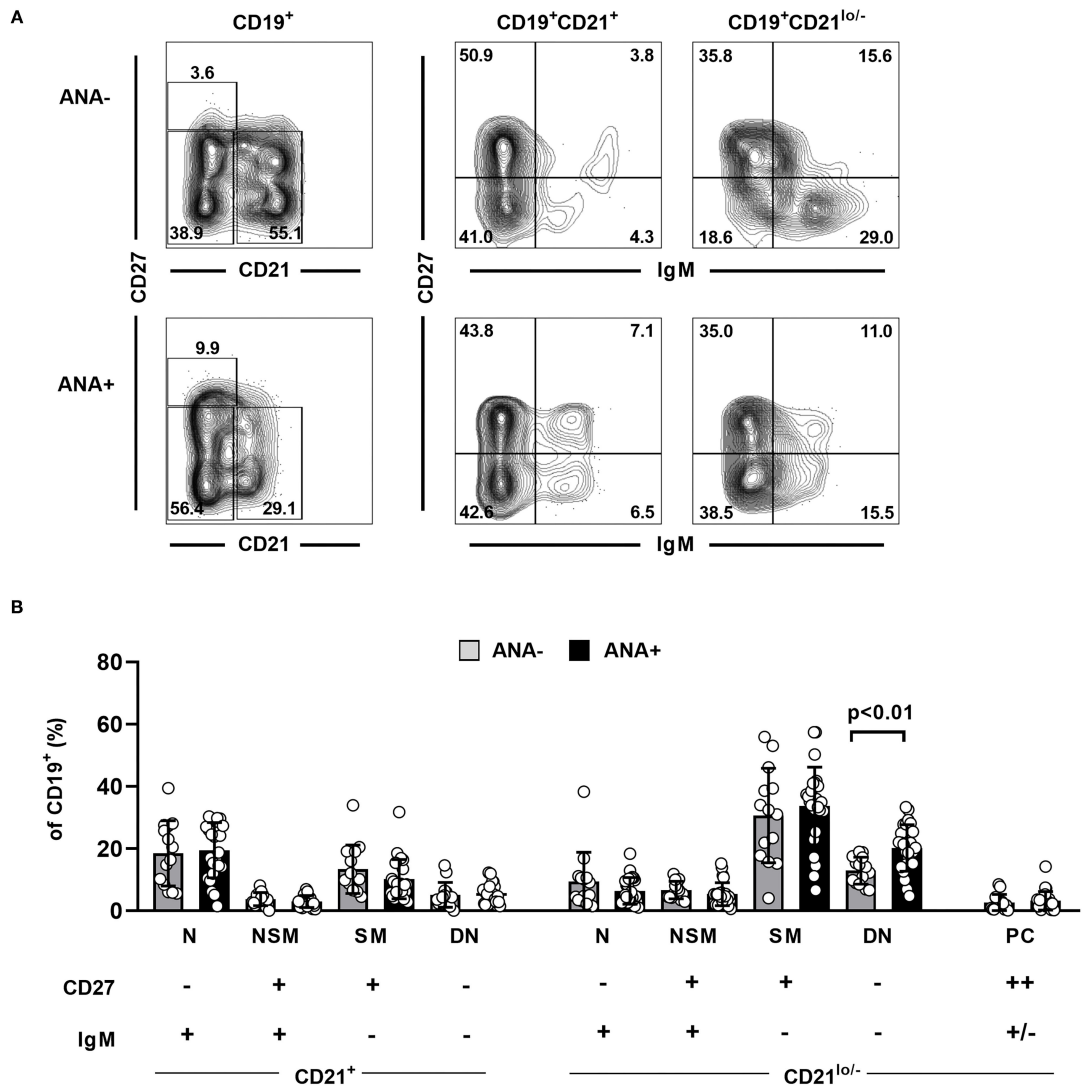
Distribution of synovial fluid B cell populations in ANA positive and negative JIA patients. **(A)** Representative contour plots showing the distribution of different B cell subpopulations as assessed by expression of CD21, CD27, and IgM in an ANA– and an ANA+ JIA patient. **(B)** Distribution of different B cell population within SF CD19^+^ B cell was analyzed using flow cytometry and compared between ANA– (*n* = 14) and ANA+ (*n* = 25) JIA patients (unpaired Student's *t*-test). Bars represent mean frequency with standard deviation and circles individual data points. N, naïve; NSM, non-switched memory; SM, switched memory, DN, double negative; PC, plasma cells.

To further verify our initial findings, we finally quantified the above-defined B cell subsets in SF samples from a cohort of 43 JIA patients and compared their distribution between ANA+ and ANA– JIA patients. Four patients with an intermediate ANA titer of 1:80 were excluded from this analysis (Supplementary Table 2). Surprisingly, we did not observe differences in the frequency of CD27^++^ plasma cells between both groups (Figure 3). Furthermore, almost none of the analyzed B cell subsets showed quantitative differences between ANA+ and ANA– JIA patients. However, the frequency of CD21^lo/−^ DN B cells was significantly higher in ANA+ than ANA– JIA patients (Figure 3B). Hence, activated CD21^lo/−^ DN B cells preferentially accumulate in the joints of ANA+ JIA patients.

## Discussion

We showed increased frequencies of transitional and switched memory B cells in the PB as well as accumulation of CD21^lo/−^ SM and DN B cells in the SF of JIA patients, the latter B cell population being particularly increased in the joints of ANA+ patients.

First, we could recapitulate recent findings describing a disturbed PB B cell pool in JIA patients, characterized by an expansion of transitional B cells as well as SM B cells (14). Expansion of transitional B cells was documented in other autoimmune diseases, already, and may be linked to defects in central and/or peripheral B cell tolerance checkpoints (24). Indeed, we described alterations of the immunoglobulin repertoire within the naïve B cell population of JIA patients that may indicate B cell tolerance defects (13). However, the alterations within the composition of PB B cell subsets in our present study were independent of the presence of ANAs and rather seem to reflect general changes seen in JIA patients. Also, the increased frequencies of SM B cells observed in this study were not associated with the presence of ANAs. Therefore, studying PB in JIA patients might not be the appropriate compartment to reveal B cell subsets potentially associated with the presence of ANAs in JIA patients.

Several reports demonstrated the presence of plasma cells and/or organized lymphoid structures in the joints or eyes of particularly ANA+ JIA patients; the latter may provide an environment for B cell differentiation at the site of inflammation (16–18). However, the B cell subsets differentiating within such structures often do not resemble those regularly found in secondary lymphoid organs. We therefore used multiparametric flow cytometry and hierarchical cluster analysis to explore SF of JIA patients for B cell subsets that preferentially accumulate in the joints of JIA patients. This approach revealed class-switched B cells with downregulation of CD21 and upregulation of CD11c and T-bet as the major B cell subset accumulating in the joints of JIA patients, a phenotype reflecting so-called CD21^lo/−^ B cells (9). Whereas, most of the SF B cell subsets analyzed in this study harbored a considerable fraction of CD21^lo/−^ B cells that also showed varying degrees of T-bet expression, CD21^lo/−^ DN B cells were the only B cell subset that showed differences between ANA+ and ANA– JIA patients: They were expanded in the SF of ANA+ JIA patients. Worthy of noting is that we could not detect significant differences in the frequency of CD27^++^ plasma cells within the SF between both JIA subgroups. Mechanistic studies from mouse models and patients with systemic lupus erythematosus suggested distinct pathways that induce differentiation of activated naïve B cells into CD21^lo/−^ DN B cells. Engagement of antinuclear B cell receptors as well as nucleic-sensing TLR7 in the presence of IFN-γ and/or IL-21 is particularly involved in this process (9–12, 25). Interestingly, we recently demonstrated an oligoclonal expansion of DN B cells accumulating in the SF of JIA patients (15). Additionally, compared to SM B cells the immunoglobulin genes of the SF DN B cell population harbored a lower load of somatic hypermutation suggesting emergence from naïve rather than “classical” memory B cells (15). It is therefore tempting to speculate whether the CD21^lo/−^ DN B cells that accumulate in the joints of ANA+ JIA patients might be enriched in antinuclear clones.

ANA-positive JIA patients seem to constitute a clinically homogenous group of patients, characterized by early disease onset, female preponderance, and high risk of uveitis (19, 20). Although it is still a matter of debate whether the group of ANA+ JIA patients constitutes a separate disease entity that also follows a distinct pathogenesis (26), CD21^lo/−^ DN B cells accumulating at the site of inflammation might reflect a distinct mechanism that is particularly involved in disease pathogenesis of ANA+ JIA patients.

The findings of this explorative study have to be seen in light of some limitations. The cross-sectional design of the study might have an impact on our observations and confounders such as current or past medication cannot be excluded. Methotrexate and TNF-α inhibitors were suggested to affect B cell differentiation in JIA patients, the latter especially impacting on memory B cell expansion (14, 27). However, since the proportion of JIA patients treated with methotrexate tended to be higher in the ANA+ than in the ANA– group (cohort 2) and none of the JIA patients was treated with TNF-α inhibitors, we do not assume that these differences in medication might explain our observation of an expanded CD21^lo/−^ DN B cell population in the joints of ANA+ JIA patients. Also, we were not able to compare the distribution of B cell populations directly between PB and SF in a large cohort of matched samples. However, this was not the aim of this study and was performed before (15). We rather sought to compare differences in B cell differentiation between ANA+ and ANA– JIA patients. Therefore, we do not see major limitations in using two independent cohorts of JIA patients for analyzing either the distribution B cells in SF or PB. However, we want to stress that the results of these two analyses should be considered separately and do not allow a direct comparison. However, the differential distribution of various B cell subsets extremely differs between both compartments and SF even contains B cell subsets that are not present in PB at all. Therefore, we would not expect that potential differences in the clinical and demographic data of both cohorts might account for the extremely heterogeneous distribution of B cell subsets observed between both compartments. We also point out that the threshold for ANA positivity is a matter of debate; however, we chose the cutoff at ≥1:160, which is consistent with previous studies (19–21).

## Conclusion

In this explorative study, we described alterations in the peripheral B cell compartment of JIA patients that, in part, are associated with the presence of ANAs. Whereas, an expansion of transitional B cells and switched memory B cells in PB seems to be associated with JIA patients in general and independent of the ANA status, expansion of CD21^lo/−^ DN B cells within the inflamed joints is a characteristic sign of ANA+ JIA patients. Further research to dissect the autoreactivity of this SF CD21^lo/−^ DN B cells in JIA patients is warranted.

## Data Availability Statement

The raw data supporting the conclusions of this article will be made available by the authors, without undue reservation.

## Ethics Statement

The studies involving human participants were reviewed and approved by Ethic Committee, University of Würzburg. Written informed consent to participate in this study was provided by the participants' legal guardian/next of kin.

## Author Contributions

HM designed and supervised the study and data analysis. JD and JF analyzed data and wrote the manuscript. GH acquired and analyzed data. HG, CH, and AH-W substantially contributed to the acquisition and analysis of the patients' samples and clinical data for the work and contributed to the writing of the manuscript. All authors contributed to the article and approved the submitted version.

## Conflict of Interest

The authors declare that the research was conducted in the absence of any commercial or financial relationships that could be construed as a potential conflict of interest.

## References

[1] Prakken B Albani S Martini A. Juvenile idiopathic arthritis. Lancet. (2011) 377:2138–49. 10.1016/S0140-6736(11)60244-4 21684384

[2] Morbach H Girschick HJ. Do B cells play a role in the pathogenesis of juvenile idiopathic arthritis? Autoimmunity. (2009) 42:373–5. 10.1080/08916930902832306 19811305

[3] Wiegering V Girschick HJ Morbach H. B-cell pathology in juvenile idiopathic arthritis. Arthritis. (2010) 2010:759868. 10.1155/2010/759868 22076178 PMC3199973

[4] Meffre E Casellas R Nussenzweig MC. Antibody regulation of B cell development. Nat Immunol. (2000) 1:379–85. 10.1038/80816 11062496

[5] Eibel H Kraus H Sic H Kienzler AK Rizzi M. B cell biology: an overview. Curr Allergy Asthma Rep. (2014) 14:434. 10.1007/s11882-014-0434-8 24633618

[6] Klein U Rajewsky K Kuppers R. Human immunoglobulin (Ig)M+IgD+ peripheral blood B cells expressing the CD27 cell surface antigen carry somatically mutated variable region genes: CD27 as a general marker for somatically mutated (memory) B cells. J Exp Med. (1998) 188:1679–89. 10.1084/jem.188.9.1679 9802980 PMC2212515

[7] Rakhmanov M Keller B Gutenberger S Foerster C Hoenig M Driessen G. Circulating CD21low B cells in common variable immunodeficiency resemble tissue homing, innate-like B cells. Proc Natl Acad Sci USA. (2009) 106:13451–6. 10.1073/pnas.0901984106 19666505 PMC2726348

[8] Isnardi I Ng YS Menard L Meyers G Saadoun D Srdanovic I. Complement receptor 2/CD21- human naive B cells contain mostly autoreactive unresponsive clones. Blood. (2010) 115:5026–36. 10.1182/blood-2009-09-243071 20231422 PMC3373152

[9] Cancro MP. Age-associated B cells. Annu Rev Immunol. (2020) 38:315–40. 10.1146/annurev-immunol-092419-031130 31986068

[10] Rubtsova K Rubtsov AV Cancro MP Marrack P. Age-associated B cells: a T-bet-dependent effector with roles in protective and pathogenic immunity. J Immunol. (2015) 195:1933–7. 10.4049/jimmunol.1501209 26297793 PMC4548292

[11] Jenks SA Cashman KS Zumaquero E Marigorta UM Patel AV Wang X. Distinct effector B cells induced by unregulated toll-like receptor 7 contribute to pathogenic responses in systemic lupus erythematosus. Immunity. (2018) 49:725–39 e726. 10.1016/j.immuni.2018.08.015 30314758 PMC6217820

[12] Myles A Sanz I Cancro MP. T-bet(+) B cells: a common denominator in protective and autoreactive antibody responses? Curr Opin Immunol. (2019) 57:40–5. 10.1016/j.coi.2019.01.002 30784957 PMC8356139

[13] Morbach H Richl P Faber C Singh SK Girschick HJ. The kappa immunoglobulin light chain repertoire of peripheral blood B cells in patients with juvenile rheumatoid arthritis. Mol Immunol. (2008) 45:3840–6. 10.1016/j.molimm.2008.05.011 18614233

[14] Marasco E Aquilani A Cascioli S Moneta GM Caiello I Farroni C. Switched memory B cells are increased in oligoarticular and polyarticular juvenile idiopathic arthritis and their change over time is related to response to tumor necrosis factor inhibitors. Arthritis Rheumatol. (2018) 70:606–15. 10.1002/art.40410 29316374

[15] Morbach H Wiegering V Richl P Schwarz T Suffa N Eichhorn EM. Activated memory B cells may function as antigen-presenting cells in the joints of children with juvenile idiopathic arthritis. Arthritis Rheum. (2011) 63:3458–66. 10.1002/art.30569 21792842

[16] Gregorio A Gambini C Gerloni V Parafioriti A Sormani MP Gregorio S. Lymphoid neogenesis in juvenile idiopathic arthritis correlates with ANA positivity and plasma cells infiltration. Rheumatology. (2007) 46:308–13. 10.1093/rheumatology/kel225 16877460

[17] Kalinina Ayuso V van Dijk MR de Boer JH. Infiltration of plasma cells in the iris of children with ANA-positive anterior uveitis. Invest Ophthalmol Vis Sci. (2015) 56:6770–8. 10.1167/iovs.15-17351 26567789

[18] Wildschutz L Ackermann D Witten A Kasper M Busch M Glander S. Transcriptomic and proteomic analysis of iris tissue and aqueous humor in juvenile idiopathic arthritis-associated uveitis. J Autoimmun. (2019) 100:75–83. 10.1016/j.jaut.2019.03.004 30885419

[19] Ravelli A Felici E Magni-Manzoni S Pistorio A Novarini C Bozzola E. Patients with antinuclear antibody-positive juvenile idiopathic arthritis constitute a homogeneous subgroup irrespective of the course of joint disease. Arthritis Rheum. (2005) 52:826–32. 10.1002/art.20945 15751057

[20] Ravelli A Varnier GC Oliveira S Castell E Arguedas O Magnani A. Antinuclear antibody-positive patients should be grouped as a separate category in the classification of juvenile idiopathic arthritis. Arthritis Rheum. (2011) 63:267–75. 10.1002/art.30076 20936630

[21] Fischer J Dirks J Haase G Holl-Wieden A Hofmann C Girschick H. IL-21(+) CD4(+) T helper cells co-expressing IFN-gamma and TNF-alpha accumulate in the joints of antinuclear antibody positive patients with juvenile idiopathic arthritis. Clin Immunol. (2020) 217:108484. 10.1016/j.clim.2020.108484 32485239

[22] Morbach H Eichhorn EM Liese JG Girschick HJ. Reference values for B cell subpopulations from infancy to adulthood. Clin Exp Immunol. (2010) 162:271–9. 10.1111/j.1365-2249.2010.04206.x 20854328 PMC2996594

[23] Corcione A Ferlito F Gattorno M Gregorio A Pistorio A Gastaldi R. Phenotypic and functional characterization of switch memory B cells from patients with oligoarticular juvenile idiopathic arthritis. Arthritis Res Ther. (2009) 11:R150. 10.1186/ar2824 19804628 PMC2787263

[24] Vossenkamper A Lutalo PM Spencer J. Translational Mini-review series on B cell subsets in disease. Transitional B cells in systemic lupus erythematosus and Sjogren's syndrome: clinical implications and effects of B cell-targeted therapies. Clin Exp Immunol. (2012) 167:7–14. 10.1111/j.1365-2249.2011.04460.x 22132879 PMC3248081

[25] Jenks SA Cashman KS Zumaquero E Marigorta UM Patel AV Wang X. Distinct effector B cells induced by unregulated toll-like receptor 7 contribute to pathogenic responses in systemic lupus erythematosus. Immunity. (2020) 52:203. 10.1016/j.immuni.2019.12.005 31940271 PMC6993874

[26] Martini A Ravelli A Avcin T Beresford MW Burgos-Vargas R Cuttica R. Toward new classification criteria for juvenile idiopathic arthritis: first steps, Pediatric Rheumatology International Trials Organization International Consensus. J Rheumatol. (2019) 46:190–7. 10.3899/jrheum.180168 30275259

[27] Roth A Glaesener S Schutz K Meyer-Bahlburg A. Reduced number of transitional and Naive B cells in addition to decreased BAFF levels in response to the T cell independent immunogen Pneumovax(R)23. PLoS ONE. (2016) 11:e0152215. 10.1371/journal.pone.0152215 27031098 PMC4816312

