# Supplementary material for: CD21lo/−CD27−IgM− Double-Negative B Cells Accumulate in the Joints of Patients With Antinuclear Antibody-Positive Juvenile Idiopathic Arthritis
Source: Front Pediatr. 2021 Apr 16;9:635815. doi: 10.3389/fped.2021.635815 (PMC8085394; doi:10.3389/fped.2021.635815)
Supplement: Supplementary file 1 [file Data_Sheet_1.docx]

Supplementary Material

**
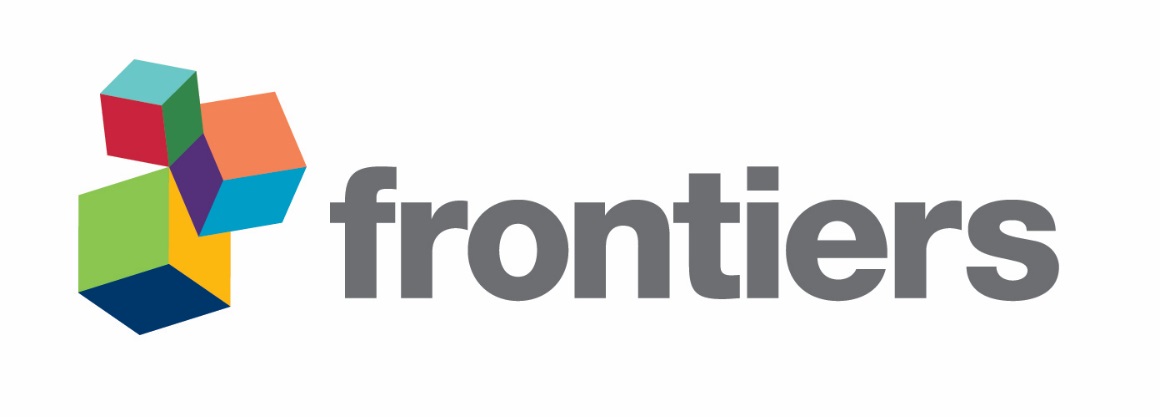
**


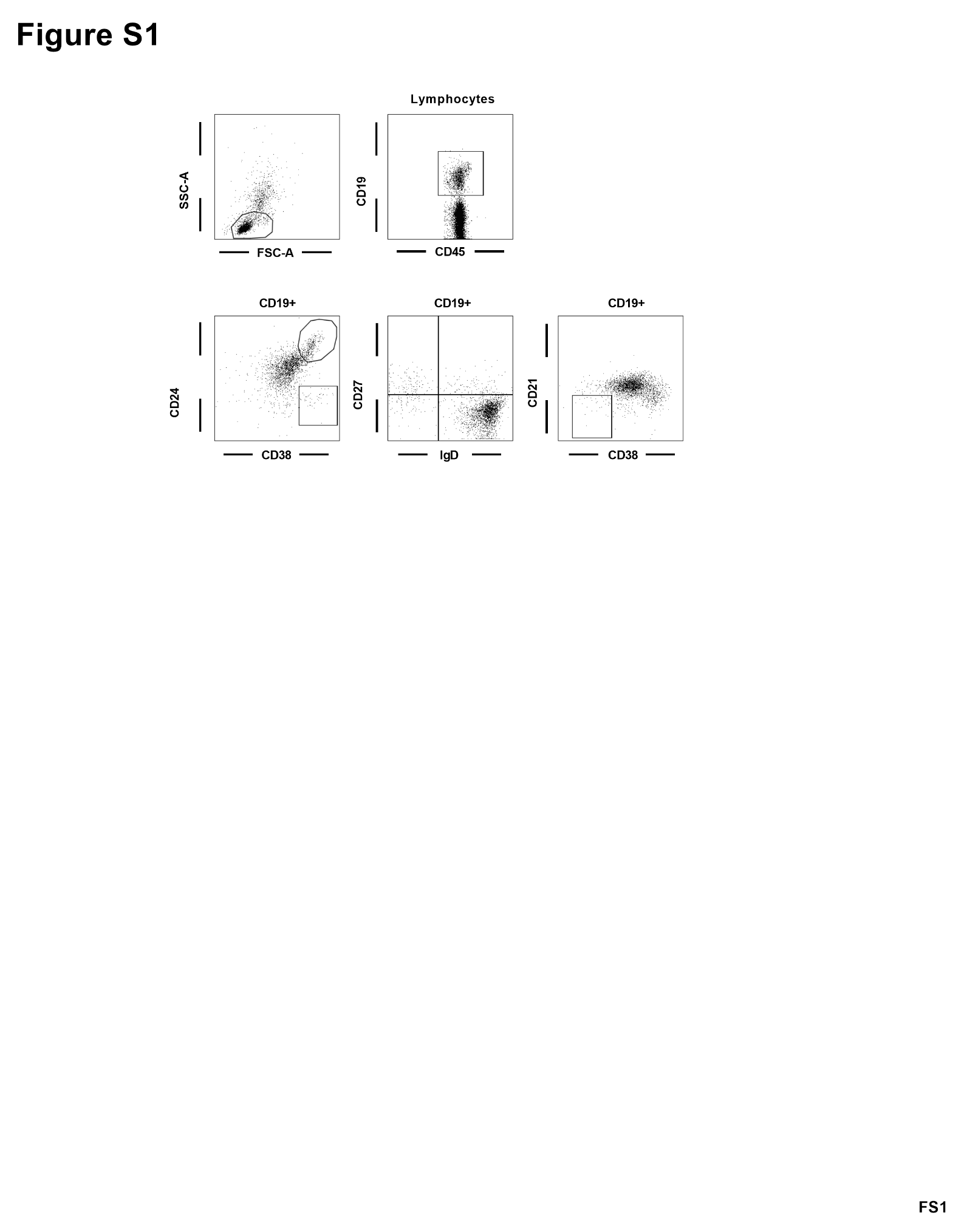


Supplemental Figure 1 – Gating strategy for the analysis of peripheral blood B cell subsets

Lymphocytes within mononuclear cells were identified based on forward-scatter (FSC) and side-scatter (SSC) characteristics as well as CD45 expression. The following populations within CD19+ B cell have been delineated: CD24^++^CD38^++^ (transitional B cells), CD24^-^CD38^++^ (plasma cells), CD27^-^IgD^+^ (naive B cells), CD27^+^IgD^+^ (non-switched memory B cells), CD27^+^IgD^-^ (switched memory B cells), CD27^-^IgD^-^ (double negative B cells) and CD21^-^CD38^-^ B cells (CD21^lo/-^). The frequency of mature naïve B cells has been calculated by subtracting the frequency of transitional B cells from that of naïve B cells.


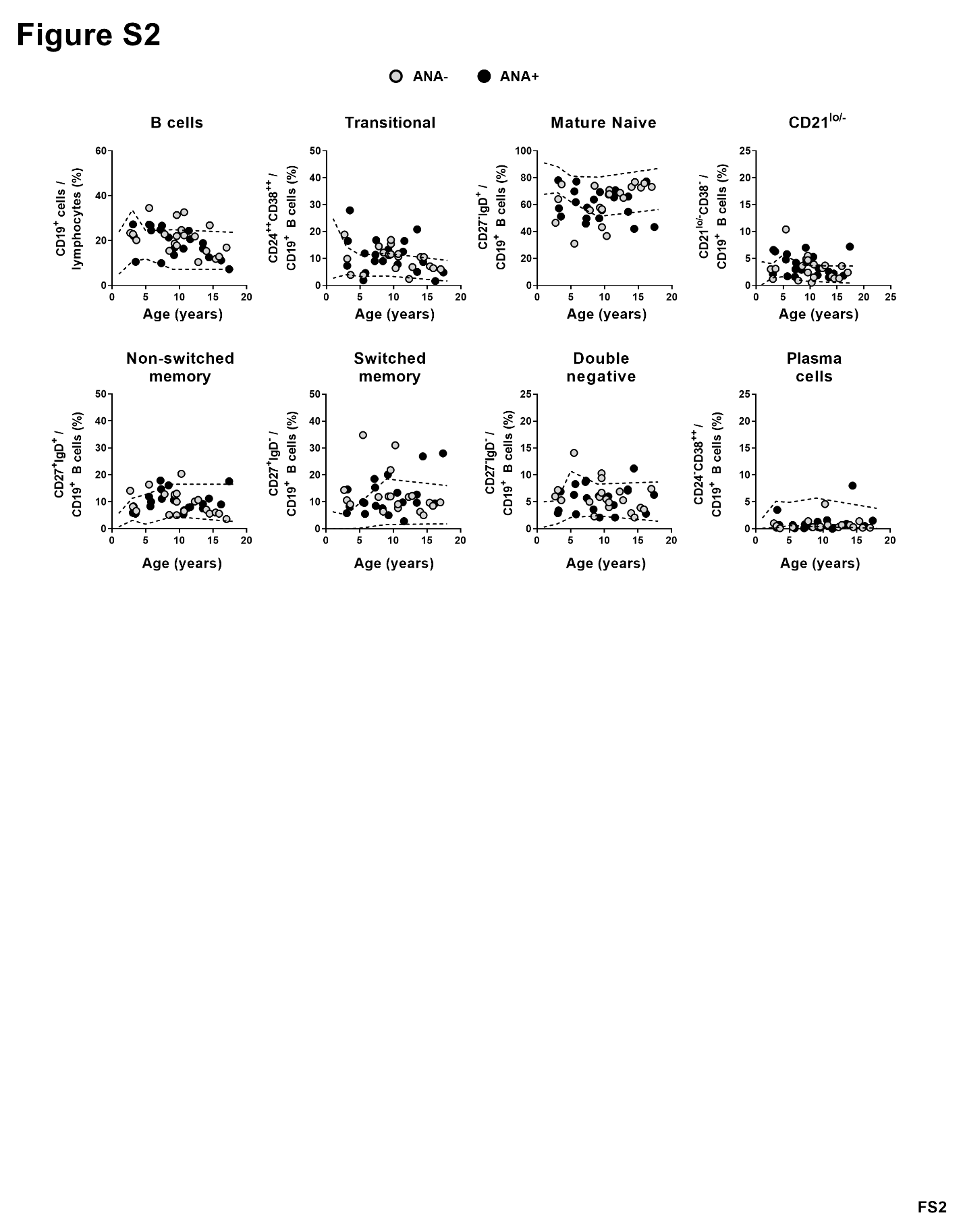


Supplemental Figure 2 – Age-dependent distribution of peripheral blood B cell population in ANA positive and ANA negative JIA patients

Data points of the frequency of different B cell populations in ANA- and ANA+ JIA patients are plotted against age. Dashed lines represent the 10^th^ and 90^th^ percentile of the distribution of each cell subset within a cohort of healthy control individuals. The proportion of data points outside the 10^th^ and 90^th^ percentile was compared between ANA- and ANA+ patients (Chi-square test). Patients with an intermediate ANA titre of 1:80 (n=5) were excluded from this analysis.

**Supplementary Table 1 – Clinical and demographic data of the studied patient (cohort 1)**

|  | All patients  (n=43) | ANA=1:80  (intermediate)  (n=4) | ANA <1:80  (negative)  (n=14) | ANA ≥1:160  (positive)  (n=25) | p-value  ANA+ vs. ANA- |
| --- | --- | --- | --- | --- | --- |
| Oligo | 32 (74.4) | 4 (100) | 11 (78.6) | 17 (68.0) | 0.71 |
| Poly | 7 (16.3) | 0 (0.0) | 2 (14.3) | 5 (20.0) | 1.0 |
| PsA | 4 (9.3) | 0 (0.0) | 1 (7.1) | 3 (12.0) | 1.0 |
| Female | 33 (76.4) | 3 (75.0) | 9 (64.3) | 21 (84.0) | 0.24 |
| Age (years) | 10.6 ± 4.9 | 14.5 2.2 | 10.3 ± 4.3 | 10.2 ± 5.2 | 0.96 |
| Age at onset (years) | 7.0 ± 4.6 | 5.5 ± 4.7 | 7.7 ± 4.6 | 6.8 ± 4.5 | 0.56 |
| Uveitis | 6 (15.0) | 0 (0.0) | 2 (14.3) | 4 (16.0) | 1.0 |
| Treatment |  |  |  |  |  |
| No medication | 10 (25.0) | 1 (25.0) | 5 (35.7) | 4 (16.0) | 0.24 |
| NSAID only | 19 (47.5) | 2 (50.0) | 5 (35.7) | 12 (48.0) | 0.52 |
| MTX and/or steroids | 14 (35.0) | 1 (25.0) | 4 (28.6) | 9 (36.0) | 0.73 |
| Biologic agent | 0 (0.0) | 0 (0.0) | 0 (0.0) | 0 (0.0) | 1.0 |

**Supplementary Table 2 – Clinical and demographic data of the studied patient (cohort 2)**

|  | All patients  (n=45) | ANA=1:80  (intermediate)  (n=5) | ANA <1:80  (negative)  (n=20) | ANA ≥1:160  (positive)  (n=20) | p-value  ANA+ vs.  ANA- |
| --- | --- | --- | --- | --- | --- |
| Oligo | 44 (97.8) | 5 (100) | 20 (100) | 19 (95.0) | 1.0 |
| Poly | 1 (2.2) | 0 (0.0) | 0 (0.0) | 1 (5.0) | 1.0 |
| PsA | 0 (0.0) | 0 (0.0) | 0 (0.0) | 0 (0.0) | 1.0 |
| Female | 27 (60.0) | 3 (60.0) | 8 (40.0) | 16 (80.0) | 0.02 |
| Age (years) | 10.6 ± 4.0 | 11.3 ± 4.1 | 12.3 ± 2.7 | 9.2 ± 4.3 | 0.01 |
| Age at onset (years) | 9.8 ± 4.1 | 4.5 ± 3.4 | 9.0 ± 5.1 | 6.9 ± 4.6 | 0.18 |
| Uveitis | 9 (20.0) | 0 (0.0) | 2 (10.0) | 7 (35.0) | 0.13 |
| Treatment |  |  |  |  |  |
| No medication | 5 (11.1) | 0 (0.0) | 3 (15.0) | 2 (10.0) | 1.0 |
| NSAID only | 22 (48.9) | 3 (60.0) | 13 (65.0) | 6 (30.0) | 0.06 |
| MTX and/or steroids | 17 (37.8) | 2 (40.0) | 4 (20.0) | 11 (55.0) | 0.05 |
| Biologic agent | 1 (2.2) | 0 (0.0) | 0 (0.0) | 1 (5.0) | 1.0 |
